# Real-time reconstruction of high energy, ultrafast laser pulses using deep learning

**DOI:** 10.1038/s41598-022-09041-y

**Published:** 2022-03-29

**Authors:** Matthew Stanfield, Jordan Ott, Christopher Gardner, Nicholas F. Beier, Deano M. Farinella, Christopher A. Mancuso, Pierre Baldi, Franklin Dollar

**Affiliations:** 1 STROBE, NSF Science and Technology Center, University of California, Irvine, CA 92617 USA; 2 Department of Computer Science, University of California, Irvine, CA 92617 USA; 3 Department of Computational Mathematics Science and Engineering, Michigan State University, East Lansing, MI 48824 USA

**Keywords:** Nonlinear optics, Ultrafast photonics

## Abstract

We report a method for the phase reconstruction of an ultrashort laser pulse based on the deep learning of the nonlinear spectral changes induce by self-phase modulation. The neural networks were trained on simulated pulses with random initial phases and spectra, with pulse durations between 8.5 and 65 fs. The reconstruction is valid with moderate spectral resolution, and is robust to noise. The method was validated on experimental data produced from an ultrafast laser system, where near real-time phase reconstructions were performed. This method can be used in systems with known linear and nonlinear responses, even when the fluence is not known, making this method ideal for difficult to measure beams such as the high energy, large aperture beams produced in petawatt systems.

## Introduction

Short pulse duration lasers ($<100$ fs) focused to relativistic intensities ($>10^{18}\text {W\,cm}^{-2}$) are used for a wide variety of applications, such as x-ray generation^1,2^, electron acceleration^3–6^, and ion acceleration^7–9^. Creating the short pulse durations used in these interactions requires the pulse to have a large spectral bandwidth along with having minimal phase shifts between the frequencies. If spectral phase differences are introduced to the pulse, significant changes to the temporal structure of the pulse can occur. The change in temporal structure could affect physical interactions, either due to a decrease in peak intensity or through complex temporal profiles introducing non-trivial interactions when used in an experiment. While knowing the temporal profile is essential for many applications, measuring the temporal profile of a high energy beam in situ is non-trivial.

Numerous techniques exist to measure the temporal profile of an ultrafast laser pulse^10–18^. This is a non-trivial task, as the fundamental theorem of Algebra prevents single spectral measurements from solving for the spectral phase relationship commonly used in tandem with the power spectrum to measure the temporal profile of an ultrafast laser pulse. This is due to non-trivial ambiguities that are introduced when converting a single power spectrum to a complex electric field, meaning no unique solutions can be found. To solve for the phase of a ultrafast laser pulse, additional information about the pulse is require to constrain the system in a way that the non-trivial ambiguities get resolved and unique solutions can be found.

A common method to gain this additional information is to measure the spectrum after a nonlinear interaction inside of some media. The nonlinear interaction is typically used to encode the phase information into a signal measurable by a square-integrable detector. Techniques such as intensity auto-correlation and frequency resolved optical gating (FROG) rely on measuring a nonlinearity induced from the interaction between two or more pulses^13^. Other techniques, such as dispersion scan (D-Scan), rely on changing the phase of the initial pulse by a known amount and monitoring how that affects the nonlinear interaction^14^. Commonly, a second harmonic mechanism is used for the nonlinear effect but other nonlinearities, such as the effects originating from the third-order term of the nonlinear electric susceptibility, $\chi _3$, have been used^10,19^. While these techniques have been highly successful, in situ measurements of high energy beams are practically difficult.

Another nonlinearity that can be used for pulse measurement is self-phase modulation (SPM). SPM is a nonlinear optical effect that occurs due to an intensity dependent index of refraction called the optical Kerr effect, which is a $\chi _3$ effect^20^. The nonlinear change in index takes the form of $n = n_0 + \gamma P(\tau )$, where $n_0$ is the linear index of refraction, $\gamma$ is the nonlinear coefficient, and $P(\tau )$ is the temporal power profile of the pulse. SPM can be modeled by the generalized nonlinear Schrödinger equation (GNLSE), which takes into account the effects of material dispersion, delayed Raman effect, and self-steepening^21^. If these effects are negligible, then the GNLSE is able to be solved analytically, taking the form of a nonlinear temporal phase shift, $E(z) = E(0)e^{i\gamma P(\tau )z}$, where *E*(*z*) is the electric field after propagating through a material of thickness z. The total amount of nonlinearity of the system is described by the B-integral, which is the integral of the nonlinear phase shift accumulated through self-phase modulation. Under the approximation that the temporal profile is constant during propagation, the B-integral simplifies to $B = \gamma P_{max}z$ with $P_{max}$ being the peak power of the pulse. In general, the inclusion of effects such as material dispersion, delayed Raman effect, and self-steepening requires the GNLSE to be solved numerically.

**Figure 1:**
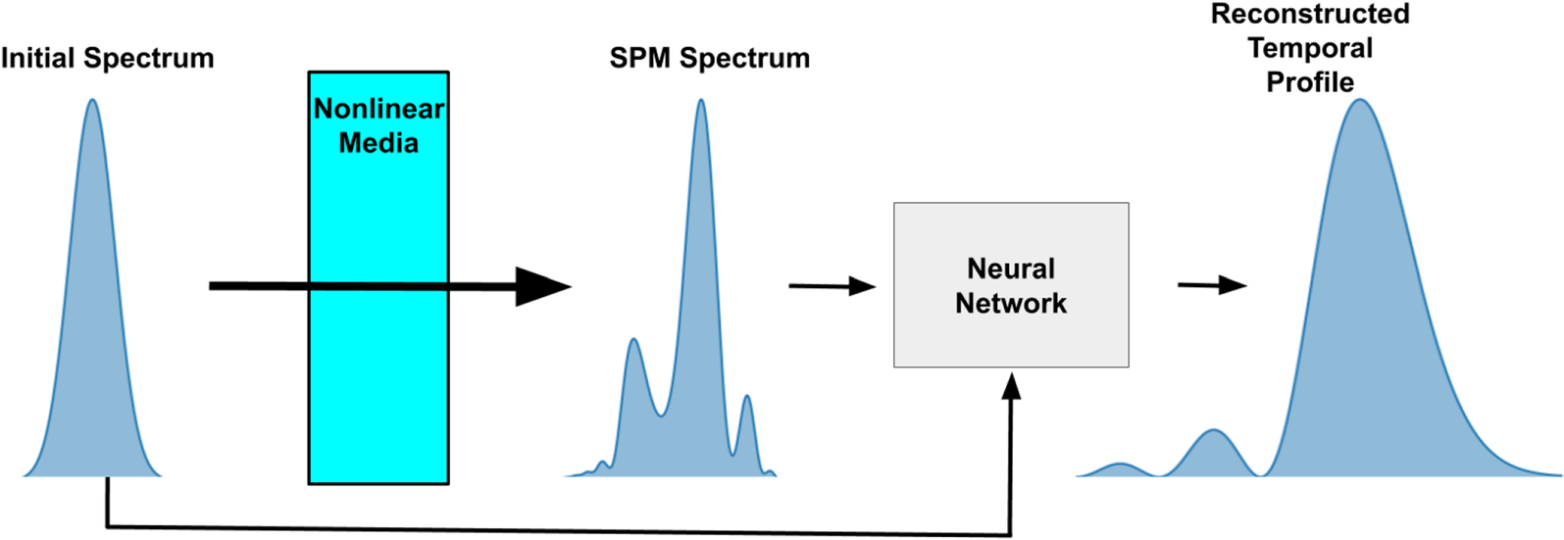
Example setup for using self-phase modulation to measure the initial temporal profile of a pulse. The initial spectrum passes through a material with a Kerr nonlinearity, causing a change in the spectrum due to self-phase modulation. A neural network takes the initial and final spectrum as inputs and extracts the initial phase and fluence of the initial pulse, allowing the initial temporal profile to be reconstructed.

Since the spectral change between the initial and SPM spectrum are directly dependent on the temporal intensity profile. This nonlinear relationship between the initial and SPM spectra can be utilized to obtain the additional information required to reconstruct both the initial spectral phase and peak power. While SPM in dispersionless media has not been explicitly proven to uniquely find the phase for all laser pulses , it has been shown it is able to accurately and robustly solve for the phase in a wide variety of laser pulses^15,16^. Specifically, the phase reconstruction does not require information about the peak power of the pulse and does not have a direction of time ambiguity (i.e. which side of the pulse is the leading edge of the pulse).

One benefit of SPMs ability to measure both the initial spectral phase and peak power is that by combining the peak power reconstruction with knowledge of the spatial profile of the laser pulse, the peak intensity of the laser pulse can also be calculated. With the usage of a high damage threshold dielectric as a nonlinear media, self-phase modulation can be performed with high intensity beams, enabling in situ pulse reconstructions of the beam for high energy beams. Commonly, the analytical solution for the GNLSE has been used in an Gerchberg-Saxton style iterative phase reconstruction algorithm, in which the phase is reconstructed from the measured spectra of subsequent thin dielectric plates^15–17^. Iterative phase retrieval methods have seen success for many-cycle laser pulses, however relying on the analytical solutions to the GNLSE limits the application to systems with negligible material dispersion. For broadband laser systems, even a small amount of material dispersion can substantially alter the way the spectrum changes from SPM, requiring the GNLSE to be solved numerically.

**Figure 2:**
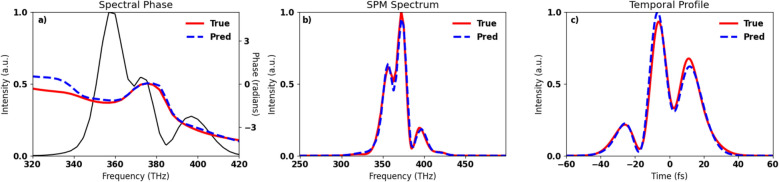
An example of a randomly generated simulated laser pulse used to train the neural network. **(A)** A randomly generated spectrum (black) centered on 374 THz along with the randomly generated phase (red) and the reconstructed initial phase (dashed blue). **(B)** GNLSE simulations based from the reconstructed initial pulse(dashed blue) and the true initial pulse (red). **(C)** Temporal profiles of the reconstructed pulse(dashed blue) and the true temporal profile (red).

Deep learning based algorithms recently have shown great promise for ultrafast laser pulse reconstructions. Deep learning has been applied to other pulse measurement techniques, such as SHG FROG and D-Scan, where neural networks replaced iterative algorithms^22,23^. Deep learning can directly learn nonlinear relationships between various features within data and map them to the desired target variables^24^. Since the information is present in the data used in the iterative phase reconstruction algorithms, deep learning can be used to directly learn the transformation between the data and the reconstructions without the need for the iterative algorithms. Since deep learning methods bypass the need for an iterative algorithm during reconstruction, deep learning approaches can be significantly faster than their iterative counterparts, enabling real-time reconstructions. Problems involving self-phase modulation have already been shown to be well suited for usage of neural networks, as neural networks have been applied to both solve the NLSE^25^ and to solve for the peak power and pulse duration of a soliton transversing an optical fiber^26^.

In this study, we demonstrate a technique to perform a robust deep learning reconstruction of the spectral phase and peak fluence of ultrafast laser pulses, which enables phase reconstruction to occur even if material dispersion is non-negligible. The reconstruction is based using neural networks trained to extract the initial phase and fluence from the relative shapes of the laser spectrum before and after self-phase modulation through a dielectric medium. The training for the neural networks is done by generating a large simulated dataset of various spectra and phases before and after self-phase modulation. By training the neural network on data generated using the GNLSE, which includes effects such as material dispersion, self-steepening, and the Raman response, the neural network is able to account of these effects in its reconstruction.

## Results

**Figure 3:**
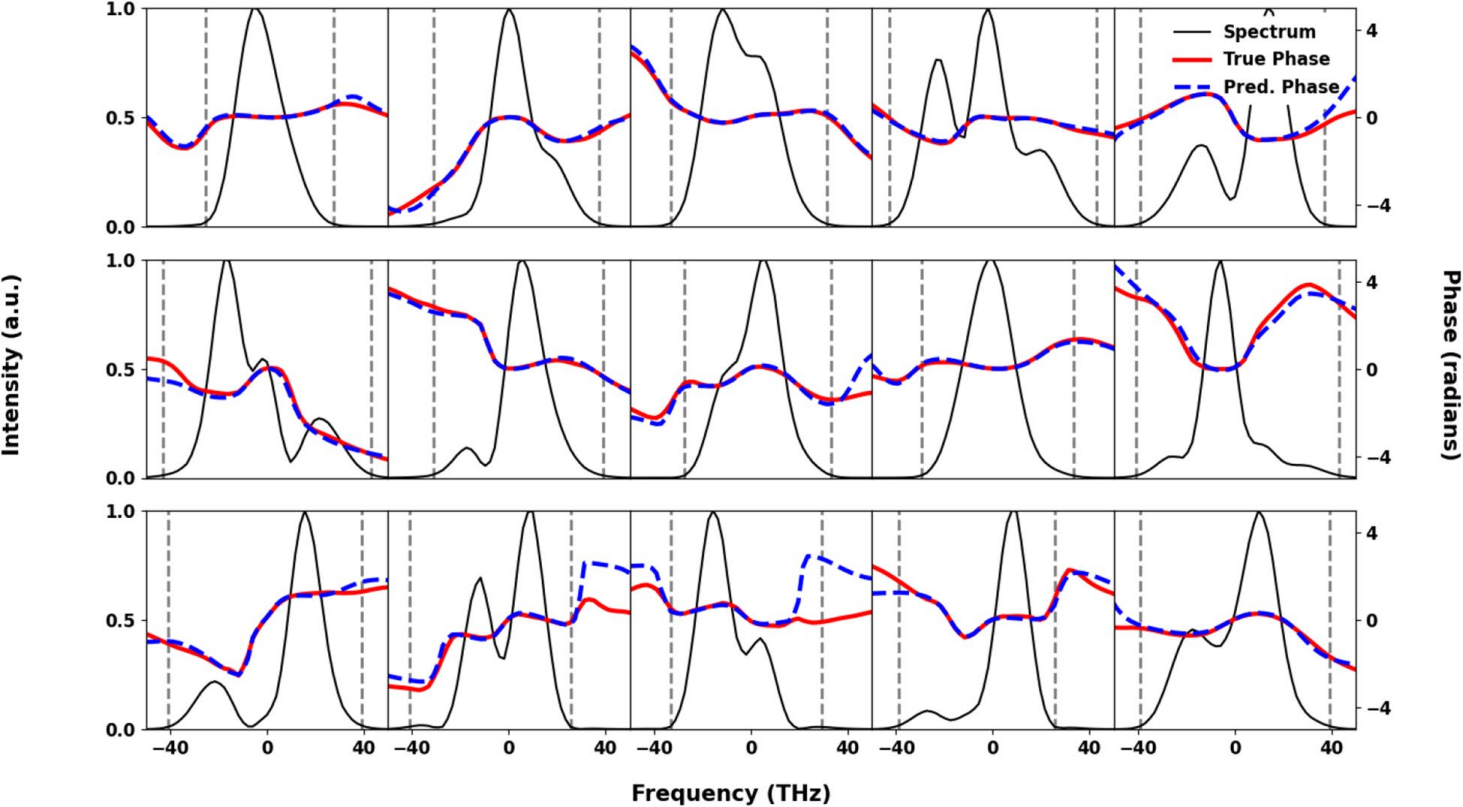
Examples of reconstructed pulses multiple examples of the variety of simulated initial spectra and phases predicted by the simulated few-cycle neural network (dashed blue) and the true phase (red). The predicted phase matches well in all regions of high spectral intensity, with disagreement only occurring in regions of near zero spectral intensity. Vertical dashed lines show location of the dB-20 spectral width.

**Figure 4:**
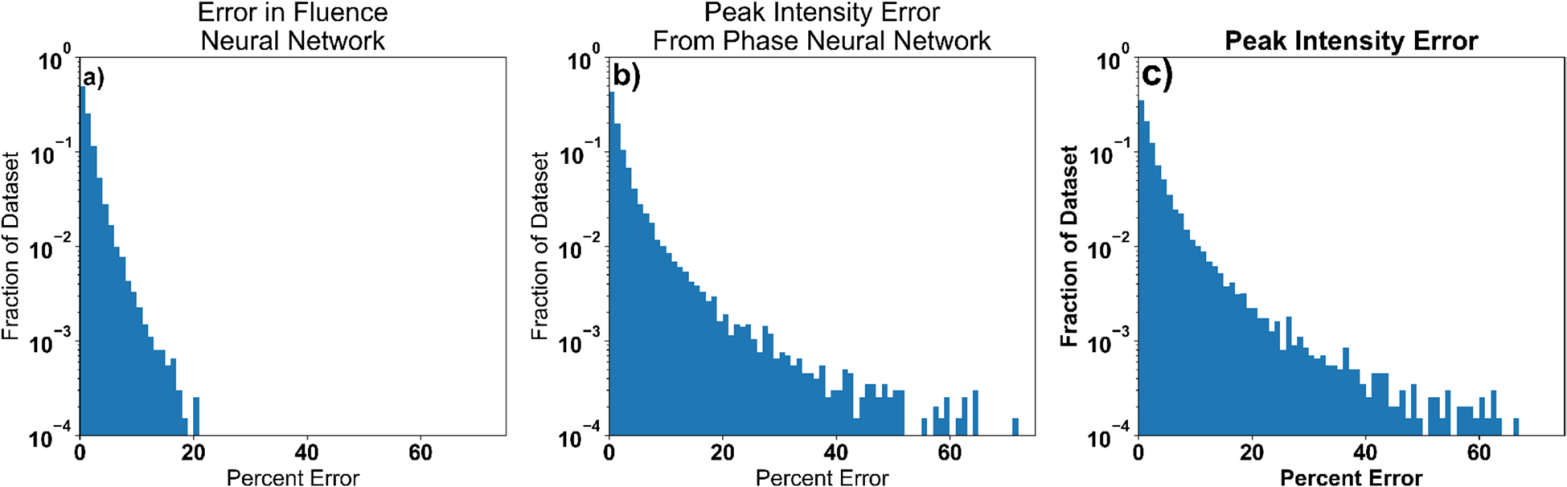
Reconstruction error in peak intensity prediction. To show the accuracy of the reconstructions of the simulated few-cycle neural networks the percent error is shown for the following systems. **(a)** Reconstruction error in the predictions for the fluence of the pulse. **(b)** Reconstruction error in the predictions for the maximum of the normalized temporal profile of the reconstructed pulse. **(c)** Reconstruction error in the predictions for the peak intensity of the reconstructed pulse using predicted fluence of the pulse.

The technique is based on predicting the phase of the initial laser pulse, as shown in Fig. 1 by using self-phase modulation in a nonlinear media and a neural network. The spectrum of the laser pulse is measured before and after sending the pulse through a known nonlinear media. The network was trained using simulated pulses to predict the spectral phase of the laser pulse from the initial and final spectrum. While simple pulses such as a Gaussian spectrum with a quadratic phase may give spectral change that could be easily human interpretable , complex pulses will have a non-trivial spectral change after undergoing self-phase modulation. While non-trivial phases would not give a human-interpretable spectral change, these complex pulses can still have the phase information retrieved by a neural network (Fig. 2).

### Broadband simulated pulse reconstruction

For the broadband simulated spectra, two separate neural networks were trained on randomly generated simulated pulses. Both networks were designed to make predictions off of the spectral measurements of the initial pulse and the pulse after SPM, with one network used to predict the initial phase of the pulses and the other network used to predict the initial fluence of the pulses. After training, 20,000 pulses withheld from the training data were run through the networks to test the accuracy of the reconstructions for previously unseen data. A set of example reconstructions are given in Fig. 3. To quantify the accuracy of the neural network on the physical qualities we are predicting, the relative reconstruction error is calculated for the fluence and the peak value of the energy normalized temporal profile. To quantify the combined accuracy of the two networks the peak value of the intensity profile is calculated.

**Figure 5:**
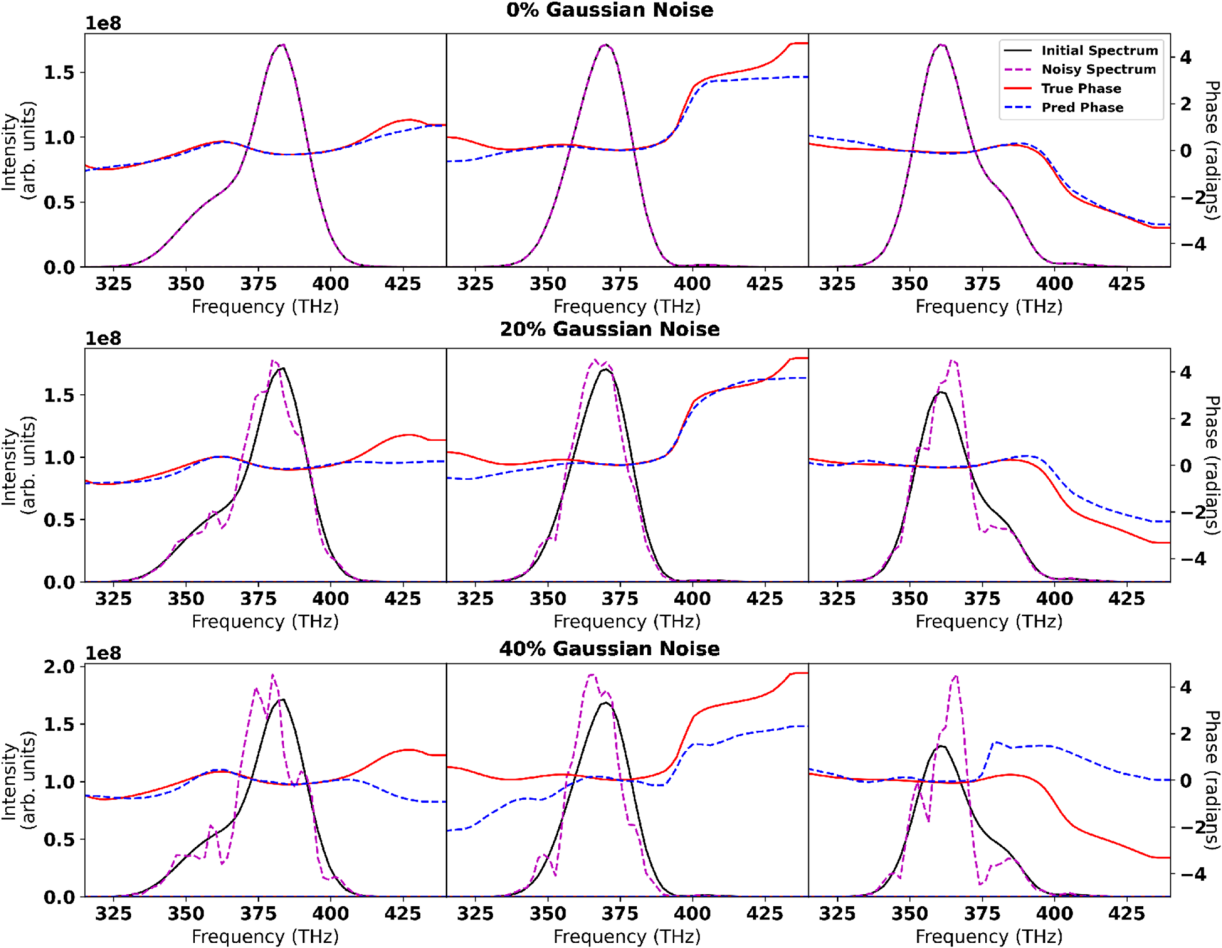
Reconstructions with Gaussian noise. To show the robustness of the phase predictions from the simulated few-cycle neural network above is three random initial spectra(black) and the noisy spectra(magenta) after adding a 0%, 20%, and 40% Gaussian noise. The true phase (solid red) is shown in comparison to the noisy phase (blue dashed) predicted from the noisy spectrum. The vertical dashed lines denote the location where the spectral intensity falls below 1% of the maximum value, outside this region the phase is ill-defined.

Comparing the reconstructed fluence to its known value provides a way to measure the accuracy of the neural network’s predictions on the physical values we are trying to predict. For $99\%$ of the pulses in the test data, the neural network was able to predict the peak fluence within an error of $<10\%$, a mean fluence reconstruction error of $1.6\%$ and a standard deviation of the fluence reconstruction error being $2.1\%$, as shown in Fig. 4. When calculating the error of the phase reconstruction, we only considered regions within the pulses dB-20 spectral width, since the phase is ill-defined and not physically meaningful outside of areas with significant power spectrum. The mean standard deviation of the predicted phase was 0.13 radians. To examine how the accuracy of the phase neural network translates into the temporal domain, the reconstruction error in the predicted maximum of the normalized temporal profile was calculated. For this calculation, $93\%$ of the pulses had a reconstruction error below $10\%$, a mean reconstruction error of $3.3\%$ and a standard deviation of the error of $6.7\%$. An example of a reconstructed pulse is shown in Fig. 2.

By combining the results from the fluence neural network with the results of the phase neural network, the temporal intensity profile can be reconstructed, including the direction of time. Using the peak temporal intensity as an estimate of the intensity reconstruction error, which has an mean intensity reconstruction error of $3.7\%$ and a standard deviation of the intensity reconstruction error of $7.1\%$ with over $90\%$ of the data set has less than $10\%$ error.

Even in the presence of noise, accurate reconstructions can be obtained. In Fig. 5, three pulses are shown with their phase reconstructions in the presence of 0%, 20%, and 40% Gaussian noise. When applied to the entire test data set, the 20% Gaussian noise caused an increase of the mean intensity reconstruction error to $5.9\%$ and a standard deviation of the intensity reconstruction error of $8.3\%$ with over $85\%$ of the data set has less than $10\%$ error. For 40% Gaussian noise the mean intensity reconstruction error to $50.1\%$ and a standard deviation of the intensity reconstruction error of $61.2\%$ with $25\%$ of the data set has less than $10\%$ error.

### Experimental pulse reconstruction

**Figure 6:**
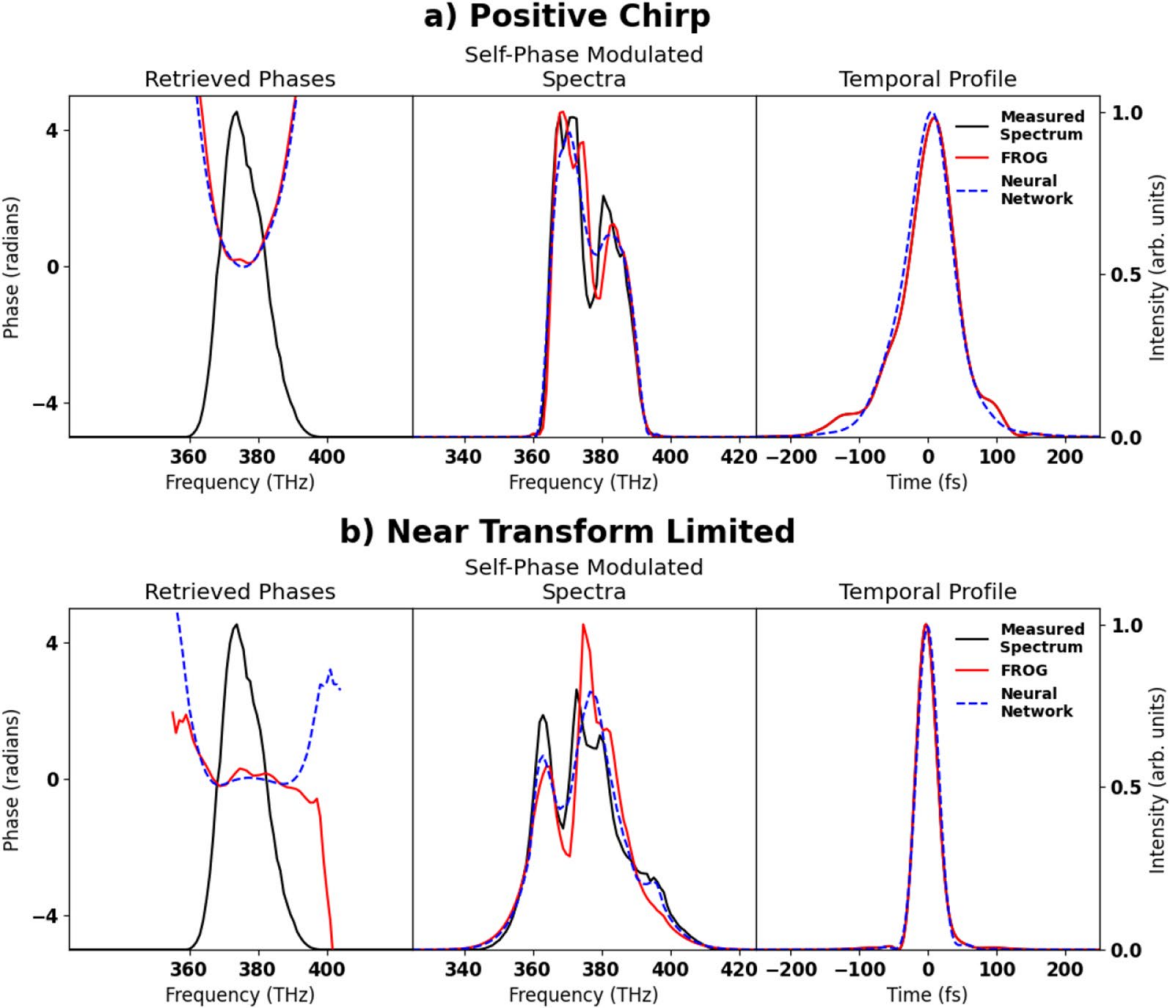
Experimental phase reconstruction phases measured from experimental data. Spectra measured after propagating through 8 mm of fused silica at Brewster’s angle. Spectra and temporal profiles are normalized to the area under the curve. **(a)** FROG reconstruction of a positively chirped laser pulse in comparison to the reconstructed results from the neural network. Self-Phase modulated spectra and temporal profile are normalized to area under the curve for ease of comparison. **(b)** FROG reconstruction of a near transform-limited pulse in comparison to the reconstructed results from the neural network.

In additional to validating the technique on broadband simulated data, we also utilized this method to reconstruct the phase of a experimental Ti:Sapphire laser pulse. This experiment was performed on a commercially available, 1 kHz repetition-rate laser system (Spectra-Physics Solstice ACE) with an energy of 6.6 mJ, beam diameter of 12 mm, central wavelength of 800 nm, and FTL pulse duration of 34 fs. The output of the laser was characterized using an SHG FROG. The nonlinear media, 8 mm of fused silica, is oriented at Brewster’s angle such that the effective propagation length after taking into account refraction is 9.6 mm. The collimated laser beam has a peak fluence of 11.7 mJ   cm$^{-2}$. Due to the large amount of material that the laser is propagating through, material dispersion will significantly impact the SPM spectrum. The spectra were taken by isolating the center of the beam with a hard aperture and sent to a fiber spectrometer utilizing a optical diffuser to minimize spectral interference from occurring inside the optical fiber.

To predict the initial phase of the laser, a new neural network was trained. While the network trained on simulated broadband pulse performed well in the previous section it has limited application to the experimental system due to the training data for that network having a bandwidth $\sim 3 \times$ larger that the experimental laser system.

Due to the laser having $<0.5\%$ root mean square energy fluctuations and a standard deviation of transform limited pulse duration $<0.75 \mathrm {fs}$, the initial laser spectrum and pulse energy can be assumed to be constant and is not needed to be included in the features given to the neural network.

The reconstruction of the experimental neural network for two separate pulses is shown in Fig. 6 and is compared to a reconstruction from a second harmonic generation FROG. The pulse duration for the positive chirped pulse predicted by the neural network was 73 fs compared to the FROGs 76 fs. The pulse duration predicted by the neural network for the near transform-limited pulse was 36 fs compared to the FROGs 35 fs pulse duration.

## Discussion

We have shown an inexpensive and easy to experimentally implement method for measuring the temporal intensity profile of the laser pulse by utilizing self-phase modulation. While two separate neural networks were trained to predict the phase for the initial pulse, this was only done to simplify the data generation process. Since a neural network is only able to predict pulses of similar structure to what it was trained on, a specifically tailored network was designed for the broadband simulated pulses and many-cycle experimental pulses. Since the majority of experimental systems output laser spectrum that are relatively consistent in spectral width this constraint does not cause significant limitations when the method is applied to experimental systems.

Due to relying only on the localized intensity of the pulse and requiring no scanning, single-shot intensity profile characterization can be performed in-situ for a collimated beam of with energies ranging from the milli-Joule to Joule level without requiring attenuation of the beam. With the usage of a imaging spectrometer and sampling the beam at various spatial locations, a 3-dimensional intensity mapping of the beam could be created. These capabilities make this technique ideal for large aperture multi-petawatt laser systems such as ZEUS^27^, which often measure the beam using a low energy sampling of the beam. When combined with the knowledge of the focal spot of an experimental system, this information would enable peak intensity of the focal spot to be calculated.

When applied to experimental data, as shown in Fig. 6, we see that both the FROG and the neural network method are in good agreement with the SPM spectrum, and the reconstructed temporal profiles are nearly identical. Since the spectral modulations tend to not contain fast varying features, high spectral resolution is not needed. For example, the neural network trained on simulated broadband pulses was trained on data with a wavelength resolution of $>4$ nm per pixel, compared to the resolution $<2$ nm resolution for the spectrometer used. The phase was able to be reconstructed using the neural network in under 10 ms on a commercially available desktop computer, implying that real-time display of the spectral phase and reconstructed temporal profile is possible.

While the assumption of a constant laser spectrum and pulse energy was able to be assumed for the experimental results discussed, environmental effects could cause a long timescale spectral drift, invalidating this assumption. For a laser system where this is a concern, a network architecture where the initial spectrum is given as an input to the neural network can be used instead of including it as an assumption of the neural network. An example of such a network is the neural networks used for the simulated few-cycle sections of this manuscript, whic has been shown to able to account for a wide range of spectral profiles and pulse energies.

OPCPA laser systems have enabled high power large bandwidth laser systems in the mid-infrared (MIR) wavelength regions. In these regions traditional silicon-based detectors no longer work, meaning forcing a reliance on more expensive InGaAs detectors. While this technique was demonstrated for wavelengths from a Ti:Sapphire laser system, the technique could be applied to the wavelengths in the MIR region. Scaling to other wavelengths would only require knowledge of material properties of the nonlinear media used along with retraining of the neural network. Being able to reconstruct the phase from only two spectral measurements enables the phase information to be readily obtainable from a field auto-correlator, meaning phase information could be reconstructed from a single power diode. Field auto-correlators are already commonly used for techniques such as Fourier transform infrared spectroscopy, making this technique simple to implement into such systems.

The ability for self-phase modulation to spectrally broaden a pulse is utilized in many pulse compression techniques to generate a few-cycle laser pulses. Modifying the neural network to predict the spectral phase after self-phase modulation would enable the reconstruction of the temporal profile after pulse compression. With this modification, the same system could be used to generate and characterize a few-cycle laser pulse.

## Conclusion

The presented pulse measurement technique shows a general technique of measuring the intensity profile of a laser pulse in single-shot applications using inexpensive and readily available components, only requiring a piece of glass and a spectrometer. By using a fully connected neural network phase reconstruction based on the generalized nonlinear Schrödinger equation is able to be done, which includes material dispersion, delayed Raman effect, and self-steepening. Since material dispersion is included in the modeling this technique is able to be used to characterize broadband laser pulses in real-time. With minor modifications, this technique enables measuring the fluence and spectral phase of the pulse across the wavefront, enabling measuring variance in the temporal profile across the beam for large aperture beams that are common at facilities such as ZEUS.

## Method

Training of the neural network occurs by running many examples of the problem through the neural network and updating the parameters of the network to find the optimum weights for predicting the desired target values. Once trained, neural networks can be used to predicted the phase of pulses similar to the training data. While neural networks can excel in extract information from data similar to what it was trained on, prediction on data vastly different from the training data can drastically hurt the performance of the network. Due to the quasi-infinite range of laser pulses possible, generating a single generalized network is non-trivial, as one would be required to ensure all possible laser spectrum and phases are present in the training data. While a single neural network may not be generalize to a complete range of spectral bandwidths, the method of generating a neural neural network still holds.

In this work, we focus on two systems of interest and train neural networks separately for each system. One system is purely simulation based and simulates a wide range of broadband spectral shapes and initial phases. The second system uses simulations to model an experimental Ti:Sapphire laser. For the experimental system, due to the stability of the laser spectra the initial spectrum is able to be assumed to reduce the number of input features into the neural network.

### Broadband simulated pulse reconstruction

#### Data generation

To generate sufficiently large data sets for training neural networks, numerical simulations of a wide range of ultrafast laser pulses with varying phases were produced. The nonlinear propagation was modeled with PyNLO, a python based 1-dimensional GNLSE solver using the split-step Fourier method^21,28^. PyNLO numerically models material dispersion, self-steepening, and the delayed Raman response. The central frequency of the simulations was set to 374.0 THz, which is the central frequency of Ti:Sapphire lasers. The material properties were based on the values for fused silica, which is a common optical glass that is able to be obtained with high optical quality and is well characterized. The material dispersion was modeled by using the second, third, and fourth order expansion curves of the Sellmeier equation for fused silica, which are 36.1 fs$^2$ mm$^{-1}$, 27.49 fs$^3$ mm$^{-1}$, and $-11.4335$ fs$^4$ mm$^{-1}$.

To ensure a representative set of phases and spectrum were present, the training data was generated from a randomly generated vector. The vector has a Gaussian envelope applied in the temporal and then spectral domains, generating a pulse with a random spectrum and spectral phase. The temporal envelope used to generate the data is 30 fs. The spectral envelope used has a width of 40 THz centered on 374 THz.

After the temporal and spectral envelopes have been applied, the peak fluence is set by randomly sampling from a uniform distribution spanning the range of 16.2 mJ cm$$^{-2}$$ to 43.2 mJ cm$$^{-2}$$. To remove the constant phase ambiguity, the spectral phase was defined to be zero at the central frequency. To remove the linear phase ambiguity, the temporal power’s central moment was set to be centered at t=0. Simulations were then ran using PyNLO inside of 1 mm of fused silica assuming a nonlinear coefficient, $$\gamma $$, of $$6 \times $$ $$10^{-8}$$ $$ (Wm)^{-1}$$. Due to the method of generating random spectra, some pulses with B-integrals $$>3$$ are generated. These pulse are not filtered out but experimental pulses with this B-integral may run into spatial effects that break the 1-dimensional assumptions made in this work^21,29^. The resulting simulations had a B-integral ranging from  0.65 to  4.25, with an average B-integral of 2.23.

The initial phase and initial spectrum were interpolated to 40 linearly space bins spanning a frequency range of 120 THz centered on 374 THz. The SPM spectrum was interpolated onto a linearly spaced vector with 100 bins and spanning the frequency range of 300 THz centered on 374 THz. After interpolation the area under the curve (i.e. the energy) for both the initial and SPM spectrum were normalized to unity to ensure the neural network is learning from the relative shape changes of the spectra. The initial and SPM spectra are then combined to create the feature vector that the neural network is trained on. A total of 1,830,000 samples were generated for the training and validation sets, with an additional 20,000 samples generated for the test set. An example pulse generated using this method is shown in Fig. 2.

#### Neural networks

**Table 1:** Hyperparameter space.

Name	Range	Parameter type	Phase	Fluence	Baseline
Batch normalization	(Yes, no)	Choice	Yes	Yes	No
Dropout	(0, 0.25)	Continuous	0.175	0	0
Learning rate	(0.00001, 0.01)	Continuous (log)	0.008	0.003	0.001
Learning rate decay	(0.5, 1)	Continuous	0.98	1	1
Number of layers	(3, 20)	Discrete	4	8	5
Number of nodes	(128, 512)	Discrete	505	360	256
Optimizer	(Adam, SGD, RMSProp)	Choice	Adam	Adam	SGD

The hyperparmaters from the optimized phase and fluence neural networks are shown in their respective columns, along with the baseline architecture.

The neural network models were trained on a dataset of 1,830,000 generated samples, initially with $70\%$ of the data in the training dataset and $30\%$ in the validation data set. The networks were trained by gradient descent (backpropagation) using the training set. The validation dataset is then used to assess their performance and make sure the networks generalize properly to previously unseen data and do not overfit the training data. A set of 20,000 additional pulses, in neither the training nor validation data set, were then re-simulated with the phases and fluence predicted by the neural networks. These final 20,000 pulses were used to perform the analysis earlier in this paper.

The input features to the neural network are the interpolated initial and SPM spectra, with a total of 140 features. The individual features of the input tend to be right-skewed, with a majority of events taking smaller scalar values and a small minority occurring in higher regions. In order to correct this we first take the log of the input features and then normalize them, by subtracting the mean and dividing by the standard deviation. The target variables are also normalized in the same fashion. Transforming the data through this process ensures all features are on the same scale.

Separate networks were trained to reconstruct the initial phase and the fluence of the pulse. The phase neural networks were trained with the targets being the initial phase of the pulse interpolated to the same 40 length frequency grid as the initial spectrum. The fluence networks were trained with the only target being the fluence of the initial pulse. All networks were implemented in Keras with a Tensorflow backend and trained on NVIDIA TITAN X GPUs.

All training samples were augmented with small amounts of Gaussian noise, ${\mathcal {N}}(0, 0.05)$, to mimic the imprecise fluctuations of experimental observations due to sources like laser fluctuations and thermal noise in silicon based detectors. This augmentation, added during training batches, also serves to prevent overfitting to the training set. Other models of experimental noise could be included by applying the noise model to the data, either during data generation or training. Training occurred over a maximum of 400 epochs. The performance of each network is characterized by calculating the mean square error loss of the predicted values compared to the target values. If the validation loss did not improve after fifteen epochs, training was terminated.

**Figure 7:**
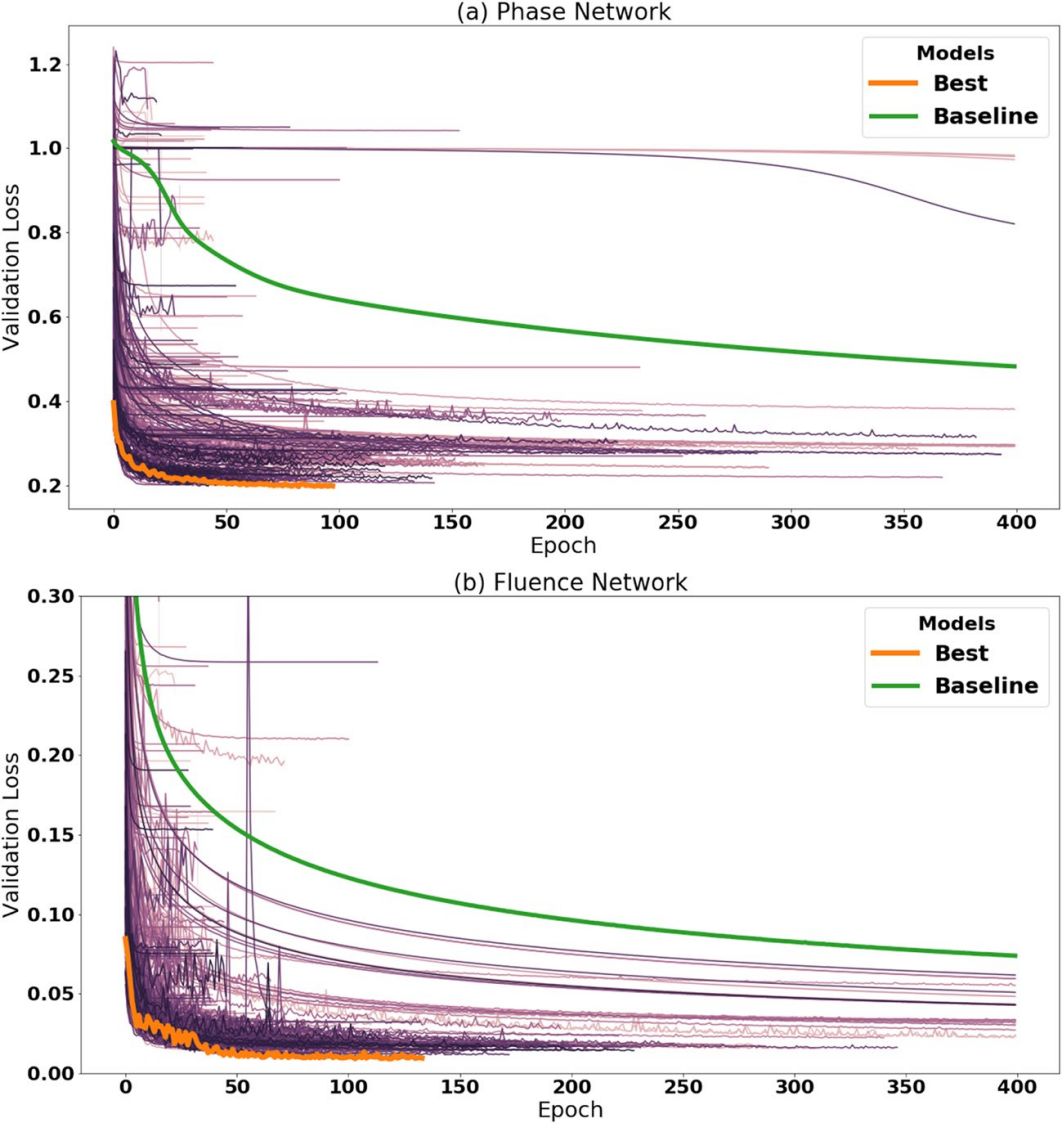
Validation loss of SHERPA trials, measured by the mean squared error, over time. Each line depicts the validation loss of a different SHERPA trial during the course of training of the simulated few-cycle networks. **(a)** Trials from phase networks with varying hyperparameters. **(b)** Trials from fluence networks with varying hyperparameters. Note: not all 500 trials are shown in each figure. Some trials with higher validation losses are left out for figure clarity. This discards 50 and 145 networks for a and b, respectively.

Building and training neural networks requires one to set many values, called hyperparameters, a priori. Hyperparameters include the number of layers, the number of nodes per layer, the kinds of activation functions, the learning rates, and the dropout rates. Dropout is a randomization procedure used during training that turns off different connections in the neural network, forcing the network to learn a more general solution which also helps avoid overfitting^30,31^.

In the experiments, the hyperparameters were optimized using SHERPA^32^, a Python software library which is compatible with Keras and other modern deep learning libraries, and has been used to effectively optimize neural networks in various scientific applications (e.g.^33,34^).

Leveraging SHERPA, a large suite of 500 models were explored using a Bayesian optimization algorithm. The Bayesian search has the advantage of learning a distribution over the hyperparameters of the network architecture, in relation to the task to be optimized. By employing this procedure we are able to evaluate a large space of possible models and test many configurations. To demonstrate the efficacy of the hyperparameter search, we compare the resulting model against an initially proposed baseline model. The baseline architecture is shown in Table 1. The optimized phase and fluence networks contained roughly 608 thousand and 970 thousand parameters, respectively. While the training time between each model varied due to the different hyperparameters, each network took approximately an hour to train.

In total, 500 network architectures were explored with differing hyperparameters for both the phase and fluence neural networks. The final architectures from the hyper-parameter search are shown in Table 1. The table displays the hyperparameters of the best performing phase and fluence network, along with the hyperparameters of the baseline network. The distribution of the validation mean squared errors (MSE) for the phase and fluence networks are shown in Fig. 7a,b respectively. These figures highlight the performance of the best optimized model compared to the initially proposed baseline network.

**Figure 8:**
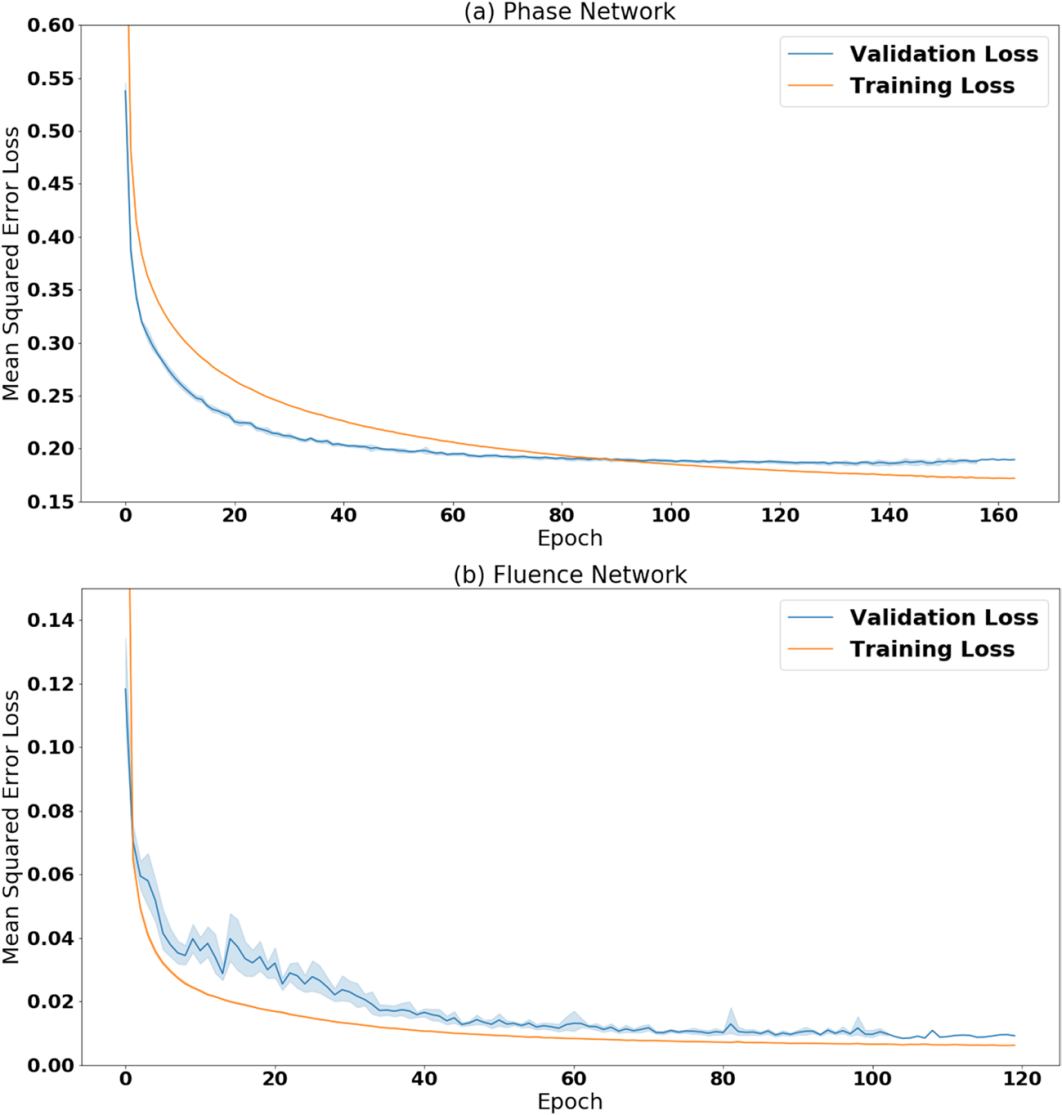
Training and validation loss, measured by the mean squared error, over time. Loss curves show the average (solid line) and one standard deviation (shaded region) for the ten folds of cross validation. **(a)** Simulated few-cycle phase network. **(b)** Simulated few-cycle fluence network.

Following the hyperparameter search, the best performing phase and fluence networks were evaluated using tenfold cross validation. During tenfold cross validation the data is randomly partitioned into ten distinct folds. Each network is then trained on nine of the folds and tested on the remaining one, and the process is repeated ten times. The mean and the standard deviation of the performance (error bars) can then be computed over the ten experiments. The results from tenfold cross validation are presented in Fig. 8a,b. These figures demonstrate consistent performance across all ten folds. We confirm that neither the phase network nor the fluence network overfits the training data by comparing the performance on the training and validation set across all ten folds. The average difference between the training and validation loss is less than 0.01 and 0.002 for the phase and fluence networks respectively.

### Experimental pulse reconstruction data generation

The simulations used to generated the training data were based on the methods discussed in the Broadband Simulated Pulse Reconstruction portion of the Methods section. Due to the dominant phase terms of the pulse being group delay dispersion (GDD) and third-order dispersion (TOD), the phase was modeled primarily as a Taylor series expansion, with random GDD, TOD, and forth-order (FOD) phase terms. The dispersion coefficient were generated from a normal distribution with the standard deviation of 10$^3$ fs$^2$, 10$^4$ fs$^3$, 10$^6$ fs$^4$ for the GDD, TOD, and FOD phase terms. To allow for minor deviations from this expansion, a random phase was generated by taking the phase of a random spectrum generated using the Fourier technique described used to generate the broadband simulated data and was added to the Taylor series phase. This Fourier phase was generated using a temporal and frequency FWHMs used were 60 fs and 50 THz with a maximum phase deviation within 25 THz of the central frequency being sampled from a normal distribution with a standard deviation of $0.5\pi$. The peak fluence of the pulse was set to match the fluence from the laser and propagated through 9.6 mm of fused silica. A total of 432 thousand simulated pulses were used for training the network.

A neural network was then trained on 432 thousand pulses total using a 80/20 split for the training and validation data sets. Due to the assumption of a single initial laser spectrum, the features used in training only needed to be based on the SPM spectrum. Both the broadened spectrum and the initial phase were interpolated to the range from 330 THz to 418 THz binned 100 linearly spaced bins. The network consisted of 8 layers with a width of 200 and was trained using a Gaussian noise of 0.1, learning rate off 0.001, drop out rate of 0.1 over 200 epochs.

## Supplementary Information

Supplementary Information.

Supplementary Video S1.

## Data Availability

The data related to this paper is available from the corresponding author upon request.
