# Supplementary material for: Real-time reconstruction of high energy, ultrafast laser pulses using deep learning
Source: Sci Rep. 2022 Mar 29;12:5299. doi: 10.1038/s41598-022-09041-y (PMC8964819; doi:10.1038/s41598-022-09041-y)
Supplement: Supplementary file 1 — Supplementary Information. [file 41598_2022_9041_MOESM1_ESM.pdf]

Real-time reconstruction of high energy, ultrafast laser pulses using deep learning

Matthew Stanfield, Jordan Ott, Christopher Gardner, Nicholas F. Beier, Deano M.Farinella, Christopher A. Mancuso, Pierre Baldi, and Franklin Dollar

Supplementary Information

Supplementary Movie 1: Experimental Real-time phase reconstruction using Ti:Sapphire Laser

The video shows a video capture of a computer program performing real-time phase retrieval on the experimental laser system detailed in the sub-section “Experimental Pulse Reconstruction”. Starting with the laser initially positively chirped, the laser’s pulse compressor was adjusted to add negative phase to the laser. This causes the pulse to continuously change from having a positive chirped, to a flat phase near the Fourier transform limit, to being negatively chirped; which is clearly observed in the phase reconstruction of the laser pulse and the experimentally measured laser spectrum after self-phase modulation.

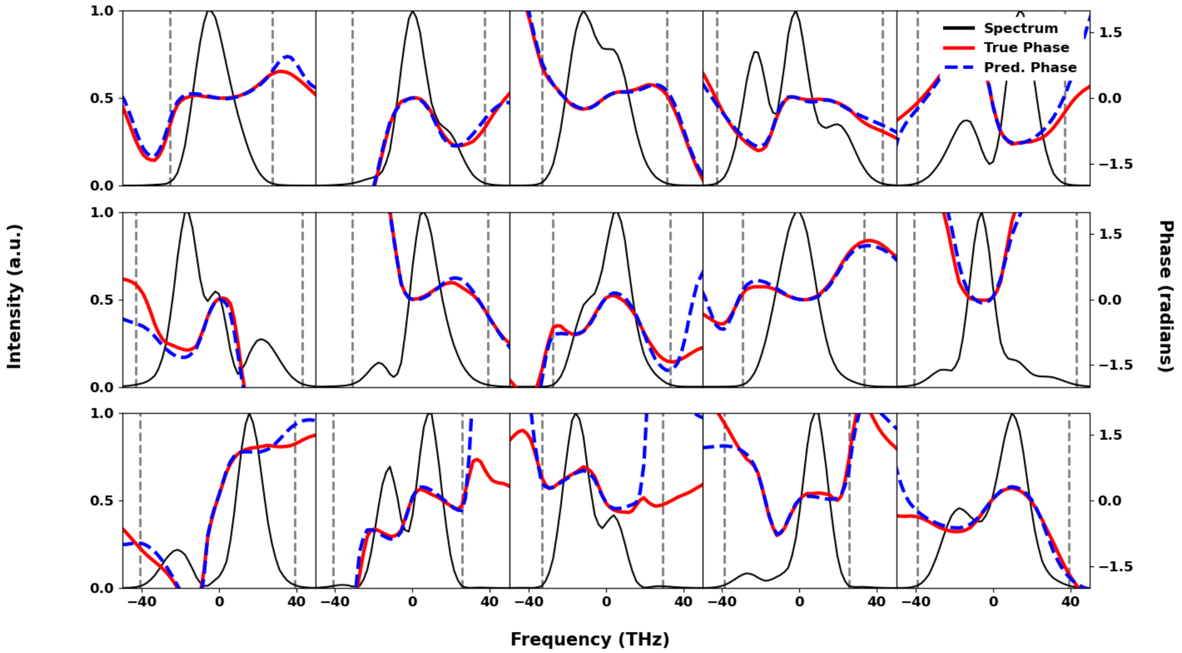

Supplementary Image 1: Phase Reconstruction from Fig. 3 with higher phase resolution

To give a better view of the phase presented in Fig. 3 of the main manuscript, Supp. Image 1 shows the same pulses with the range of phases being zoomed in to provide better phase resolution.
